# Requirement of Innate Immunity in Tumor-Bearing Mice Cured by Adoptive Immunotherapy Using Tumor-Draining Lymph Nodes

**DOI:** 10.1155/2015/170852

**Published:** 2015-06-10

**Authors:** John Ammori, Khaled Hamzeh, Hallie Graor, Julian Kim

**Affiliations:** Division of Surgical Oncology, Department of Surgery, University Hospitals Case Medical Center and Case Comprehensive Cancer Center, Cleveland, OH 44106, USA

## Abstract

*Background.* The purpose of this study was to determine the cellular effectors of both the adoptively transferred cells and the tumor-bearing host that participate in the antitumor response to adoptive immunotherapy using culture-activated tumor-draining lymph nodes (TDLNs). *Methods.* TDLNs harvested from mice with 4T1 carcinoma cells were fractionated to derive the L-selectin^low^ subpopulation and activated* ex vivo* prior to *in vitro* cytokine release assays and adoptive transfer into BALB/c mice bearing 3-day established subcutaneous tumors. Tumor-bearing recipients were SCID (lacking T, B, and NK cells), Rag2 deficient (lacking T and B cells), and wild-type BALB/c mice. *Results.* Culture-activated L-selectin^low^ 4T1 TDLN from BALB/c mice secreted significant levels of interferon-gamma in response to 4T1 but not control tumor cells *in vitro*. CD4 cells within the adoptively transferred effector cell population contributed significantly to the antitumor effect *in vivo*. Culture-activated L-selectin^low^ TDLNs from BALB/c wild-type mice were able to cure Rag2 deficient but not SCID mice bearing 4T1 subcutaneous tumors, suggesting a requirement of NK cells within the innate immune system of the tumor-bearing host during the antitumor response. *Conclusions.* These results identify the cellular effectors involved in tumor regression following adoptive transfer and demonstrate the requirement for intact innate immunity within the tumor-bearing host.

## 1. Introduction

Adoptive transfer of immune cells into a cancer-bearing host has been shown to be efficacious both in animal models and in human trials. Several approaches have been used, such as the use of tumor-infiltrating lymphocytes, dendritic cell vaccines, lymphokine-activated killer cells, vaccine-primed lymph nodes, chimeric antigen receptor-modified T cells, and tumor-draining lymph node (TDLN) cells [1–13]. Several groups have explored adoptive immunotherapy (AIT) using TDLN, which has shown significant therapeutic activity against a variety of murine tumors [4, 6, 8, 12–19]. Lymph nodes (LN) draining progressively growing subcutaneous tumors contain antigen-specific preeffector T cells which have been sensitized to tumor cells but are not fully functional effector T cells. Following* ex vivo* activation with anti-CD3 and culture with interleukin-2 (IL-2), these preeffector cells acquire tumor-specific effector function against established tumor* in vivo* [18]. Despite the polyclonal nature of the cell culture with anti-CD3 and IL-2, the resultant T cells mediate* in vivo* antitumor effect only against the tumor with which they were stimulated* in vivo* [12]. Though demonstrating* in vivo* therapeutic effect, these cells do not exhibit cytotoxic activity against tumor targets* in vitro* in some experimental models while they do in others [12, 20]. Activated TDLNs secrete interferon-*γ* (IFN-*γ*) in response to exposure to tumor cells* in vitro* [5, 15, 20–22].

To optimize therapeutic efficacy, the number and type of cells transferred are important. Fresh TDLN cells transferred to a cancer bearing animal result in no tumor response.* Ex vivo* expansion with anti-CD3 and IL-2 not only expands the number of cells by several folds but also activates the cells for therapeutic effect. In addition, T cells with low expression of the T cell homing receptor L-selectin (L-selectin^low^) represent a group of potent tumor reactive T cells from within the total TDLN cell population [21, 23–25]. Transferred L-selectin^low^ cells isolated from TDLN had a 30-fold greater therapeutic efficacy than unfractionated TDLN cells [23]. Cytokine secretion is induced by exposure of activated T cells to tumor cells* in vitro* [23, 24]. Transfer of only L-selectin^high^ cells had no therapeutic effect [23, 24]. L-selectin^high^ cells have been shown to act as suppressor T cells that can prevent L-selectin^low^ T cell antitumor activity [26]. The experiments in this study utilize L-selectin^low^ T cells from TDLN.

Freshly harvested lymph nodes contain an array of immune cell types. After expansion and activation with anti-CD3 and IL-2, the cell population is enriched with T cells. During* in vivo* tumor growth, sensitization of CD8 T cells in the TDLN occurs independently from sensitization of CD4 T cells [13]. During* in vitro* expansion and activation with anti-CD3 and IL-2, CD8 cells require the helper function of CD4 cells [13]. In some experimental models, transfer of either CD4 or CD8 cells activated TDLN cells induced* in vivo* tumor regression, while in other models CD4 alone or CD8 alone was therapeutically ineffective [15, 21, 23]. The role of the host immune system in adoptive T cell therapy is not entirely clear. Regulatory T cells suppress tumor reactive T cells, and lymphodepletion with chemotherapy has been used as an approach to enhance adoptive T cell therapy [27].

The purpose of this study was to determine the cellular effectors that play a central role in the antitumor efficacy of adoptive immunotherapy using TDLN in an animal model. In this report, we demonstrate that treatment with L-selectin^low^ T cells from TDLN draining 4T1 mammary tumors cause tumor regression. The CD4 cells demonstrate more therapeutic activity than the CD8 cells. Natural killer (NK) cells and/or other components of the innate immune system in the tumor-bearing host are necessary for antitumor effect of the transferred TDLN cells.

## 2. Materials and Methods

### 2.1. Adoptive Immunotherapy Protocol

**Figure 1:**
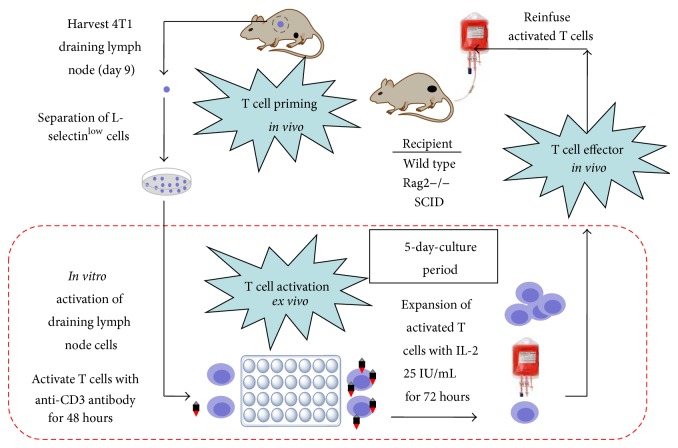
Tumor vaccination, T cell activation, and adoptive transfer.

The 4T1 is a 6-thioguanine-resistant cell line that was selected from a tumor cell line derived from a single spontaneously arising mammary tumor in a BALB/c3H mouse. All animal studies were approved by the Institutional Animal Care and Use Committee. Eight 12-week-old female BALB/c mice were inoculated subcutaneously with 1 × 10^6^ 4T1 mammary tumor cells in the bilateral flanks. After 9 days of tumor growth, draining inguinal lymph nodes were surgically obtained and single cells were prepared and activated in Complete Media on 24-well plates (Costar, Cambridge, MA) with immobilized anti-CD3 monoclonal antibody (clone 145-2C11) at a density of 4 × 10^6^ cells/2 mL/well for 48 hours. Following activation, cells were harvested and expanded with 25 U/mL IL-2 for 72 hours. Activated T cells were resuspended in Hank's Balanced Salt Solution (HBSS) and adoptively transferred in 1 mL intravenously via the tail vein to mice bearing 3-day established 4T1 subcutaneous tumors.  Figure 1 demonstrates this protocol.

### 2.2. Immunomagnetic Selection of TDLN Subsets

Freshly harvested 4T1 TDLNs were subjected to immunodepletion using immunomagnetic beads and columns (Miltenyi Biotec). CD62L (L-selectin) microbeads were used to derive the L-selectin^low^ prior to anti-CD3/IL-2 culture activation. A second round of immunoselection was performed using CD4 and CD8 microbeads in select experiments.

### 2.3. Tumor-Bearing Recipient Mice

Activated 4T1 TDLNs were adoptively transferred into 3-day established tumor-bearing recipient BALB/c mice. The recipient mice included BALB/c wild-type mice, BALB/c SCID (lacking T and B lymphocytes as well as NK cells), and BALB/c Rag2 deficient mice (lacking functional T and B lymphocytes). All mice were purchased from Jackson Laboratories, Bar Harbor, ME. Mice were followed and tumor size was assessed.

### 2.4. Tumor-Specific Activated T Cell Cytokine Release as Measured by ELISA

Subcutaneous tumors were established in BALB/c mice using 4T1 cells as well as the murine renal cancer cell line Renca. Activated T cells were cocultured with irradiated 4T1 or Renca cells from freshly harvested subcutaneous tumors at an effector T cell to tumor target (E : T) ratio of 2 : 1 in 24 well plates. Supernatants from activated T cell cultures were analyzed for interferon- (IFN-) *γ* using ELISA (R&D Systems). IFN-*γ* release was measured and reported as pg/mL/10^6^ T cells/24 hrs.

## 3. Results

### 3.1. Isolation of L-Selectin^low^ T cells

It is known that naive T cells express high levels of CD62L (L-selectin^high^). L-selectin expression is lost during the effector phase and activated T cells are L-selectin^low^. In order to characterize the T cell population obtained from TDLN, the following experiments were performed. TDLNs were harvested from the inguinal region of BALB/c mice inoculated with 4T1 mammary cancer cell line. The cell population was characterized prior to culture activation. Approximately 30% of the TDLN cell population prior to culture activation was L-selectin^low^, suggesting antigen-priming of T cells within TDLN* in vivo* (Table 1). Both the L-selectin^low^ and the L-selectin^high^ populations consisted of a mix of CD4 and CD8 T cells as well as B lymphocytes as indicated by the B220 antigen. After 5-day culture activation of the L-selectin^low^ cell population, the final culture consists predominantly of a mixture of CD4 and CD8 cells at a 3 : 2 ratio. In addition, approximately 40% of the final culture contained B cells.

### 3.2. L-Selectin^low^ TDLNs Demonstrate Tumor-Specific Cytokine Secretion

**Figure 2:**
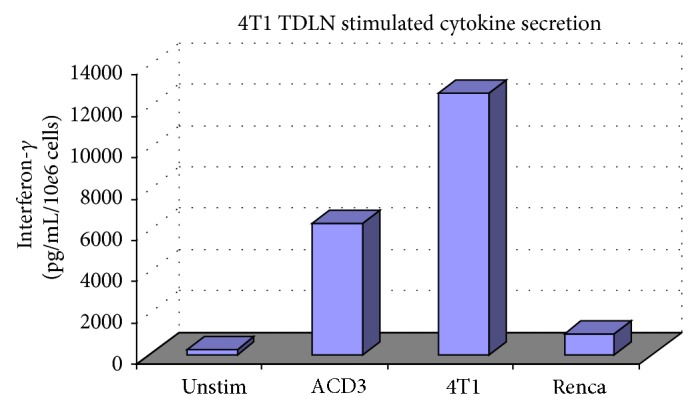
Activated TDLNs were cocultured alone, with immobilized anti-CD3, irradiated 4T1, or irradiated Renca tumor cells for 24 hours.

The next experiment sought to measure the secretion of interferon-*γ* (IFN-*γ*) by effector T cells, which is a major contributor to the antigen-specific therapeutic response in AIT. Secretion of the cytokine IFN-*γ* by 4T1 TDLN was examined under multiple conditions to assess tumor-specific reactivity* in vitro* (Figure 2). 4T1 TDLNs were fractionated to derive L-selectin^low^ effector T cells which were then activated in culture for 5 days with anti-CD3 and IL-2. The activated T cells were then cultured for 24 hours under 4 different conditions: (1) TDLN alone as a negative control, (2) TDLN with immobilized anti-CD3 as a positive control, (3) TDLN with irradiated 4T1, and (4) TDLN with the irradiated renal cancer cell line Renca, which is MHC-compatible with 4T1 (MHC haplotype: H-2K^d^). 4T1 TDLN exhibited tumor-specific reactivity to 4T1 cells compared to Renca cells as measured by secretion of IFN-*γ*. These data suggest that the culture-activated T cells obtained from lymph nodes draining 4T1 mammary cancers in this animal model are primed specifically against tumor antigens from the 4T1 cell line.

### 3.3. Therapeutic Effectiveness of Adoptive Immunotherapy and the Role of the Recipient's Immune System

**Figure 3:**
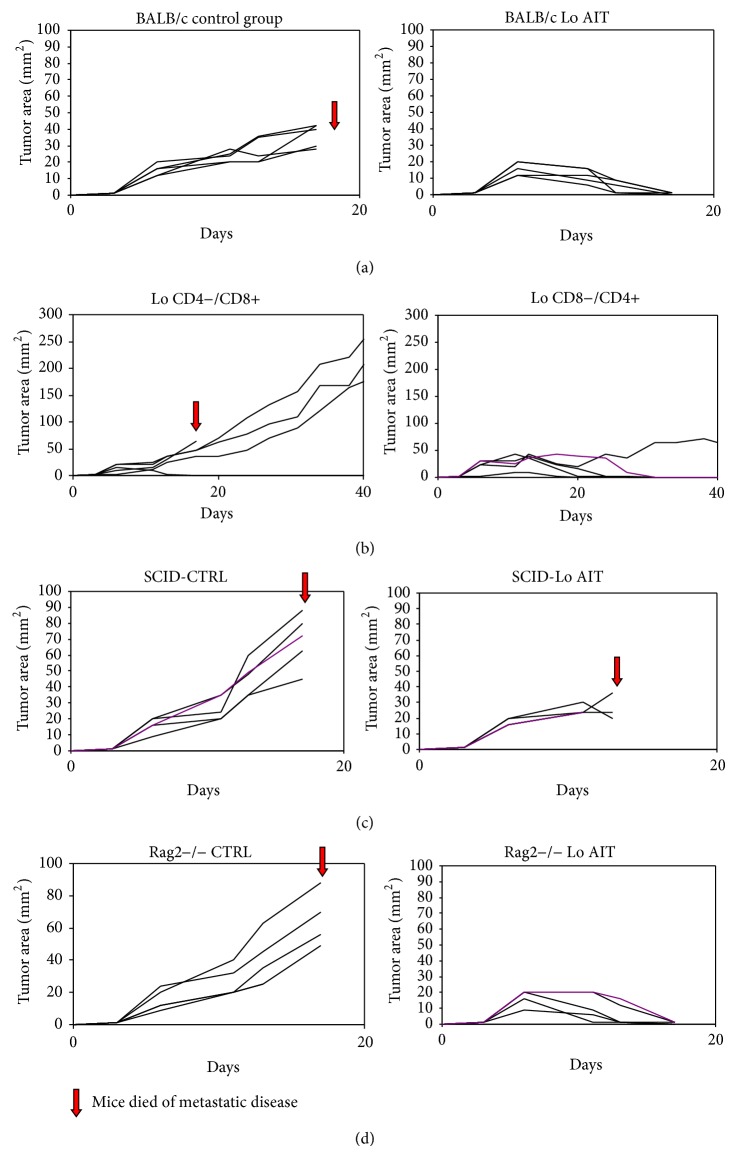
(a) 4T1 TDLNs transferred into BALB/c mice. BALB/c mice bearing 3-day established 4T1 subcutaneous tumors treated with 4T1 TDLNs (Lo AIT) were cured of tumor while those treated with HBSS (control) died of metastatic disease. Each line represents one mouse (*n* = 5). (b) CD4/CD8 depleted TDLNs into BALB/c mice. BALB/c mice with 4T1 subcutaneous tumors were treated with 4T1 TDLNs depleted of CD4 or CD8 cells. Therapeutic efficacy of transferred TDLN appears to be primarily related to intact CD4. Each line represents one mouse (*n* = 5). (c) 4T1 TDLNs transferred into SCID mice. SCID mice bearing 3-day established 4T1 subcutaneous tumors treated with 4T1 TDLNs and control mice all died, suggesting the need for some immune competence in the tumor-bearing mice for tumorigenicity of transferred TDLNs. Each line represents one mouse (*n* = 5). (d) 4T1 TDLNs transferred into Rag2−/− mice. Rag2−/− mice bearing 3-day established 4T1 subcutaneous tumors were treated with HBSS (CTRL, *n* = 4) or 4T1 TDLNs (Lo AIT, *n* = 5). Results suggest that NK cells within the tumor-bearing host are necessary for therapeutic activity of transferred TDLN. Each line represents one mouse.

The next series of experiments sought to determine the therapeutic activity of TDLN in BALB/c mice* in vivo*. First, we evaluated the transfer of L-selectin^low^ cells into immunocompetent wild-type BALB/c recipients bearing 3-day established tumors (Figure 3(a)). All 5 mice treated with control injections died of metastatic disease within 20 days. In contrast, all 5 mice treated with 4T1 TDLN demonstrated shrinkage of tumor with ultimate disappearance and cure. This finding shows that AIT using 4T1 L-selectin^low^ TDLN completely treats established 4T1 mammary cancers in this model.

In order to determine the contribution of the CD4+ and CD8+ T cell subtypes in the adoptively transferred cells to the overall therapeutic activity, the following experiment was performed. BALB/c mice bearing 3-day established tumors were treated with adoptive transfer of TDLN depleted of CD4+ or CD8+ cells (Figure 3(b)). Mice treated with T cells containing only CD8+ or CD4+ cells demonstrated tumor response and survival to 40 days, suggesting that both T cell subtypes play a role in antitumor activity. The majority of mice treated with only CD4+ T cells demonstrated cure, suggesting that the therapeutic efficacy appears to be primarily related to CD4+ T cells in this model system.

Finally, the contribution of the tumor bearing host's immune system on the therapeutic effects of adoptive T cell therapy was determined using recipient mice with specific immune deficiencies. 4T1 tumors were established subcutaneously in SCID mice, which lack T cells, B cells, and NK cells. All tumor-bearing SCID mice died of metastatic disease regardless of treatment with control injection or TDLN which was curative in immunocompetent wild-type BALB/c mice (Figure 3(c)). This finding suggests that some level of immune competence in the host is necessary for therapeutic efficacy of adoptively transferred TDLN. 4T1 tumors were also established subcutaneously in Rag2 deficient mice, which lack only T and B cells. Tumor-bearing Rag2 deficient mice that were administered control treatment died of metastatic disease, whereas those treated with TDLN were cured (Figure 3(d)). This finding suggests that NK cells or other components of the innate immune system within the tumor-bearing host are necessary for anti-tumor efficacy of transferred TDLN in this model.

## 4. Discussion

In this study, we demonstrated that AIT using culture-activated L-selectin^low^ T cells derived from 4T1 TDLN cause tumor regression in a syngeneic murine model of mammary cancer. Culture-activated 4T1 TDLN cells demonstrated tumor-specific T cell reactivity evidenced by secretion IFN-*γ* in response to 4T1, but not Renca tumor cells,* in vitro*. Both CD4 and CD8 T cells were necessary for maximal antitumor effect, but CD4 cells demonstrated more therapeutic activity than CD8 cells. Using mice deficient in components of the immune system, we found that NK cells and/or other components of the innate immune system in the tumor-bearing host are necessary for antitumor effect of the transferred TDLN cells in this model.

Modulating the immune system in order to treat cancer is a treatment approach which has been expanding in clinical use recently. Adoptive immunotherapy with the activation of immune cells* ex vivo* followed by cell transfer into the tumor-bearing host has been a method used for over 20 years, with moderate clinical success [28]. Many different strategies had been used both in the clinical and research settings. This includes infusion of tumor-infiltrating lymphocytes (TILs), lymphokine-activated killer cells (LAKs), vaccine-primed lymphocytes, T cells genetically engineered to express tumor-specific antigen receptors, and T cells with chimeric antigen receptors (CARs). T cell therapy has been combined with infusion of the T cell growth factor IL-2, as well as nonmyeloablative leukoreductive therapy using chemotherapy with or without total body radiation. The combined therapy using nonmyeloablative chemotherapy, T cell infusion, and systemic IL-2 has demonstrated 20% complete response rate and 70% overall response rate in melanoma patients [29].

Isolating T cells from tumor-draining lymph nodes provides another source for cellular immunotherapy. Lymph nodes draining a growing tumor contain tumor-sensitized pre-effector T cells.* Ex vivo* activation and expansion with anti-CD3 and IL-2 differentiate these preeffector cells into effector T cells [12, 13]. Numerous animal studies have shown the therapeutic efficacy of TDLN immunotherapy, and* in vitro* studies have shown tumors cytotoxicity of human TDLN cells to human melanoma cells [5, 8, 12, 13, 17, 18, 20, 21].

Down regulation of L-selectin on T cells is an early event in the response of antigenic stimulation. L-selectin^low^ T cells comprised 30% of our TDLN population, which is consistent with other reports [24]. Adoptive transfer of isolated L-selectin^low^ T cells has demonstrated therapeutic antitumor efficacy in a sarcoma animal model, while isolated infusion of L-selectin^high^ T cells was ineffective [24]. In addition, interferon-*γ* production in response to tumor exposure is significantly higher in L-selectin^low^ versus L selectin^high^ T cells, suggesting this population contains more tumor reactive T cells [21]. Furthermore, infusion of L-selectin^high^ cells from TDLN have been shown to block effector responses in adoptive immunotherapy protocols using TDLN in an animal model [26]. The use of L-selectin^low^ T cells is an attractive methodology for immunotherapy protocols as it allows for isolation of a population of highly reactive antitumor effector cells which can then be expanded tremendously. Although adoptive immunotherapy using L-selectin^low^ cells from TDLN in other animal models has used sublethal radiation for conditioning, the therapy described in our experimental model did not use radiation or IL-2 as an adjunct. This has potential relevance for clinical application of this therapy administered in the absence of conditioning with radiation or IL-2, which both have their toxicities.

Although both adoptively transferred CD4 and CD8 cells were required for tumor regression, CD4 cells were more effective. CD8+ cytotoxic T cells are exclusively used in many adoptive immunotherapy models as these are known to have effector function after activation. In a sarcoma model using anti-CD3 activated TDLN, CD8 cells caused tumor regression while CD4 cells did not result in tumor regression but instead provided a helper function to CD8 cells that could be replaced with exogenous IL-2 [13]. However, in a similar sarcoma model using TDLN which were anti-CD3/anti-CD28 activated, CD4 cells were more potent effector cells than CD8 cells [15]. Similarly, another report showed that L-selectin^low^ CD4 cells caused tumor regression [23]. It has also been reported that progressive tumor growth in syngeneic mice leads to independent sensitization of both TDLN L-selectin^low^ CD4 and CD8 cells, with CD4 cells being more effective against 3-day established intracranial and pulmonary tumors and CD8 cells more effective against 10-day established tumors [25]. In the current study, tumor regression was most effective with both CD4 and CD8 cells. CD4 cells demonstrate direct cytotoxicity and likely provide a supportive role by secretion of cytokines to enhance CD8 cells cytotoxicity. Similar to previous reports, the antitumor activity did not require exogenous IL-2.

Though not investigated in the current study, it is possible that adoptively transferred B cells played a role in mediating tumor regression. B cells predominantly perform antigen-presentation and antibody production. Although T cells are the predominant effector cells, B cells can also function as effector cells. In a mouse model of pulmonary metastases, activated TDLN B cells mediated significant tumor regression. This response was enhanced by the addition of activated TDLN T cells [30]. Activated B cells in adoptive immunotherapy of solid tumors have demonstrated regression of sarcoma, melanoma, and breast cancer [30, 31]. The mechanism for B cell tumor activity is unknown, but the B cells may support the antitumor activity of the activated T cells in culture. In addition, B cells' role as antigen-presenting cells may enhance the host's T cell response. The adoptive transfer of activated B cells specific for 4T1 into hosts bearing 4T1 tumors resulted in the induction of systemic T cell immunity to 4T1 [31].

NK cells are effector lymphocytes of the innate immune system which were found to be an integral component within the host for tumor response in the studied model system. NK cells do not require antigen recognition and are able to lyse tumor cells without prior stimulation [32–34]. They recognize target cells through activating receptors on the cell surface. Mechanisms involved in tumor cytotoxicity include perforin/granzyme mediated cytotoxicity, death receptor mediated apoptosis, and IFN-*γ* secretion [35]. NK cells play a role in tumor immunosurveillance as evidenced by the fact that mice deficient in NK cells are more susceptible to methylcholanthrene-induced sarcomas [36]. In addition, NK cells mitigate the growth of lymphomas in mice lacking perforin and *β*2 microglobulin [37]. Furthermore, NK cells prevent pulmonary and peritoneal metastases and peritoneal dissemination in murine models [38, 39]. Other experimental models have demonstrated the cooperation of the innate and adaptive immune systems. For example, in a murine model of intraperitoneal tumors of mesenchymal origin, complete tumor rejection was dependent on both T cells and NK cells [40]. Similarly, in a murine model of subcutaneous and lung tumors, treatment of subcutaneous tumors using photodynamic therapy led to a CD8 and NK dependent inhibition of growth of untreated lung tumors [30]. In treatment models using dendritic cell-based adoptive immunotherapy, the interaction of NK cells with dendritic cells and T cells has been shown to be important for antitumor responses [38]. In the model used in the current study, there is evidence of cooperation of the innate and adaptive immune systems. NK cells may have a direct cytotoxic effect upon exposure to the tumor. Alternatively, cytokine release from CD4 and/or CD8 cells may trigger an NK cell response.

In conclusion, the present study demonstrated that (1) adoptive transfer of culture-activated 4T1 TDLN cells into immunocompetent syngeneic tumor-bearing hosts results in tumor regression, (2) culture-activated 4T1 TDLN cells secrete interferon-gamma in response to 4T1 but not Renca tumor cells* in vitro*, demonstrating the presence of tumor-specific T cell reactivity, (3) adoptive transfer of L-selectin^low^ subpopulations into immunocompetent tumor-bearing hosts suggests that CD4 cells within the culture-activated TDLN mediate a significant proportion of the antitumor effect, and (4) adoptive transfer of culture-activated 4T1 TDLN into SCID and Rag2 deficient mice suggests that NK cells within the tumor-bearing host are critical to the observed antitumor effect. These results demonstrate the interaction between cellular components of both the adaptive and innate immune systems in this model of AIT using TDLNs. Strategies to enhance the interaction between adoptively transferred T cells and resident NK cells or innate immunity may result in improved antitumor therapeutic efficacy.

## Figures and Tables

**Table 1:** FACS analysis of baseline 4T1 TDLNs and after culture activation of the L-selectin^low^ subpopulation.

	CD4	CD8	THY1.2	L-sel	CD11b	CD11c	B220
	(% (SD))	(% (SD))	(% (SD))	(% (SD))	(% (SD))	(% (SD))	(% (SD))
Day 0: all cells	36.5	19.5	56.4	29.7	3.6	1.2	42.6
	(7.0)	(3.7)	(4.5)	(11.6)	(0.9)	(1.1)	(1.1)

Day 0: CD62L-low	27.74	6.0	35.6	1.4	6.7	3.5	55.3
	(9.5)	(2.2)	(9.1)	(0.6)	(2.1)	(3.2)	(16.4)

Day 0: CD62L-high	46.3	24.1	66.7	25.6	1.73	2.12	39.0
	(6.3)	(4.8)	(9.5)	(9.9)	(1.3)	(3.7)	(9.3)

Day 5: CD62L-low	33.3	20.9	59.6	1.6	1.6	0.9	46.8
	(4.4)	(8.5)	(6.8)	(0.8)	(1.1)	(0.2)	(13.4)

4T1 TDLNs contain approximately 30% of cells that demonstrate downregulation of CD62L suggestive of antigen-priming *in vivo.* The L-selectin^low^ subpopulation contains higher proportions of CD4 prior to culture activation and consists predominantly of Thy1.2+ lymphocytes after *ex vivo* expansion. Each experiment was performed 5 times.
